# Assessment of maternal knowledge of solar exposure and vitamin D in the neonatal period

**DOI:** 10.1590/1984-0462/2024/42/2023040

**Published:** 2023-12-11

**Authors:** Sandra Mara Witkowski, Catarina Pfitzer, Emanueli Rudolf, Gabriela de Souza, Aline Didoni Fajardo, Flávia Maestri Nobre Albini, Fabricio Sbroglio Lando, Marco Otilio Duarte Rodrigues Wilde

**Affiliations:** a Universidade do Vale do Itajaí, Hospital Infantil Pequeno Anjo, Itajaí, SC, Brazil.; b Hospital Infantil Pequeno Anjo, Itajaí, SC, Brazil.

**Keywords:** Solar radiation, Vitamin D, Neonate, Skin cancer, Radiação solar, Vitamina D, Neonato, Câncer de pele

## Abstract

**Objective:**

To assess mothers’ knowledge on sun exposure related to serum vitamin D levels in the neonatal period.

**Methods:**

Observational, analytical and cross-sectional study, carried out from August 2020 to May 2021 through a questionnaire directed to mothers of newborns, in a maternity hospital in Southern Brazil.

**Results:**

From 141 interviewees, 132 (93.6%) believe it is important to expose the neonate to sun, 101 (71.6%) think this exposure can increase vitamin D levels, 86 (61%) received such information from a doctor, 108 (76.6%) believe there are no risks of sun exposure, 88 (62.4%) claim it isn´t necessary to use any kind of protection, 96 (68.1%) said that only exposure to the sun was necessary to maintain adequate levels of vitamin D during the neonatal period. Only two mothers (1.4%) claim that you should not exposure the neonate to the sun, and only one (0.7%) stated that sun expose can cause skin problems.

**Conclusions:**

Most mothers lack satisfactory knowledge about sun exposure related to serum vitamin D levels in the neonatal period. The need to inform and clarify the population about sun exposure during this period is remarkable, in addition to disseminating the proper way to maintain serum levels of vitamin D.

## RESUMO

**Objetivo:**

Avaliar o conhecimento das mães acerca da exposição solar relacionada com níveis séricos de vitamina D no período neonatal.

**Métodos:**

Estudo observacional, analítico e transversal, realizado de agosto de 2020 a maio de 2021 por meio de questionário dirigido às mães de recém-nascidos, em uma maternidade no sul do Brasil.

**Resultados:**

De 141 entrevistadas, 132 (93,6%) acreditam ser importante expor o lactente ao sol no primeiro mês de vida, 101 (71,6%) acham que essa exposição aumenta os níveis de vitamina D, 86 (61,0%) receberam tal informação de um médico, 108 (76,6%) acreditam que expor o neonato ao sol não causa riscos para a saúde, 88 (62,4%) acham que não é necessário usar proteção contra radiação solar ao expor o neonato ao sol, e 96 (68,1%) afirmaram que apenas a exposição ao sol basta para manter os níveis adequados de vitamina D durante o período neonatal. Apenas duas mães (1,4%) afirmaram que não se deve expor o neonato ao sol e uma (0,7%) que a exposição solar pode causar problemas de pele.

**Conclusões:**

A maioria das mães não possui conhecimento satisfatório acerca da exposição solar relacionada aos níveis séricos de vitamina D no período neonatal. É notável a necessidade de informar e esclarecer a população sobre a exposição solar nesse período, além de disseminar a maneira adequada de manter os níveis séricos de vitamina D.

## INTRODUCTION

Vitamin D is a hormone that regulates calcium and phosphorus metabolism. One of its main functions to maintain bone mineralization, therefore it is essential for proper skeletal growth in children and adolescents.^1^ It is obtained through sun exposure, feeding or using dietary supplements.^2^ Its production depends on the amount of UVB that reaches the skin and therefore it is influenced by skin pigmentation, use of sunscreen, type of clothing, season of the year, day period and geographical latitude.^3^

Although society has extensive notion on the benefits of vitamin D, little is known about the consequences of exposing the newborn to the sun in an inadequate way. It is known that exposure is cumulative, and the first 20 years of life are determinants for future risks of skin cancer, especially melanoma.^4,5^ There is no safe level for sunbathing to be recommended, so it should be avoided in infants under six months and limited in other young age groups.^6^ Parents have an essential function in the photoprotection of their children, through behavior and correct habits from their first days of life. However, there is a general lack of information on this subject.^3^ The present study assesses mothers’ knowledge about sun exposure aimed at mainly at maintaining adequate serum levels of vitamin D in the neonatal period.

## METHOD

This is a cross-sectional study, carried out from August 2020 to May 2021, approved under number 4.189.572 on 8 March 2020 by the Research Ethics Committee. This study was held at the Hospital and Maternity in Itajaí, Santa Catarina (298 births/month), and mothers of newborns having the following characteristics were enrolled: 0–7 days of life, clinically stable, close to discharge, weighing more than 1.5 kg and/or more than 32 weeks of gestational age. Incomplete reports and primigravida mothers were excluded as they had not undergone high or prior experience guidance. The initial number of mothers was 150, all admitted through the public health system; nine incomplete reports were excluded, ending with a total of 141 interviewees.

The questionnaire was developed by the authors. After filling out the questionnaire, the mothers were given a folder containing information and guidance on correct sun exposure in the neonatal period, as well as on how to maintain the desirable levels of vitamin D. The questionnaire was applied by the collection team before mothers received any counseling on the subject.

The results of quantitative variables were described by mean and standard deviation. For categorical variables, frequency and percentage were presented. Data were analyzed using the Stata/SE v14.1computer program (StataCorp LP, USA).

## RESULTS

From the total of 141 interviewees, the average + standard deviation — SD (variation) age was 28.9±7.4 (15–45), with 2.9 living children per mother; 39.7% had completed elementary school and only 10.6% had completed higher education.

Regarding maternal knowledge about sun exposure related to serum vitamin D levels during the neonatal period, data are described in Tables 1 and 2.

**Table 1.** Maternal knowledge about sun exposure.

Variables	n (%)
Do you think it is important, in the first month of life, to expose your baby to the sun?	
No	3 (2.1)
Yes	132 (93.6)
I don´t know	6 (4.3)
Were you instructed by a doctor to expose to the sun any of your children, in the first month of life?	
No	13 (9.2)
Yes	116 (82.2)
Any other person	12 (8.5)
Do you think that exposure to the sun in the first month of life could cause any risk to the baby’s health?	
No	108 (76.6)
Yes	17 (12.1)
I don´t know	16 (11.3)
Do you think that exposing your baby to the sun in the first month of life can increase the chance of having any skin disease in the future?	
No	108 (76.6)
Yes	12 (8.5)
I don´t know	21 (14.9)
How long should a baby be exposed to the sun in the first month of life?	
<10 minutes	24 (17.1)
10–30 minutes	114 (80.8)
Any other	1 (0.7)
Should not be exposed	2 (1.4)
Do you think some kind of sun protection is necessary when exposing your baby to the sun?	
No	88 (62.4)
Yes	49 (34.7)
I don´t know	2 (1.4)
Did not answer	2 (1.4)
If yes, what kind of protection should be used when exposing the baby to the sun?	
Sunscreen	11 (22.5)
Clothes	11 (22.5)
Accessories	12 (24.3)
Any other	3 (6.2)
Should not expose	2 (4)
Don´t know	10 (20.5)

**Table 2.** Maternal knowledge about sun exposure and vitamin D.

Variables	n (%)
Where you instructed by a doctor to expose one of your children to the sun to obtain VD?	
No	32 (22.7)
Yes	86 (61)
Any other person	23 (16.3)
Have you heard that exposing the baby to the sun can improve serum VD?	
No	26 (18.4)
Yes	115 (81.5)
Could a lack of vitamin D cause any health problems for your child?	
No	11 (7.2)
Yes	63 (45)
I don´t know	66 (47.1)
Did not answer	1 (0.7)
Do you think that exposure to the sun in the first month of life could improve VD?	
No	16 (11.3)
Yes	101 (71.6)
I don´t know	24 (17)

VD: vitamin D.

**Figure 1.**
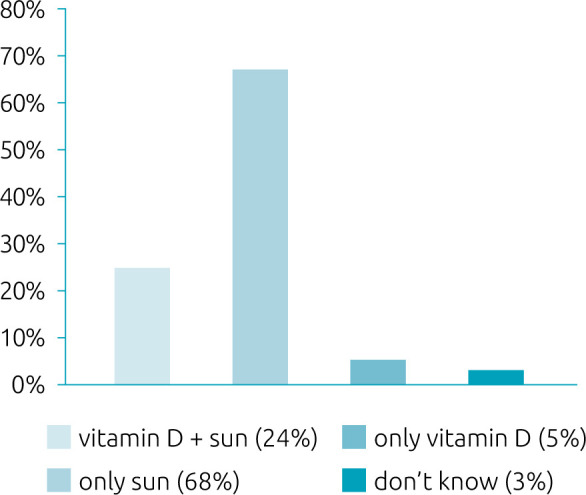
Mothers’ knowledge about how to maintain adequate levels of vitamin D during the neonatal period.

Among those mothers who answered affirmatively that vitamin D deficiency could cause health problem to their child (45%), jaundice was the reported damage (n=6), followed by bone problems (n=5), calcium deficiency (n=2) and anemia (n=2). Only one (0.7%) mother mentioned skin problems associated with sun exposure. The reports on how to maintain adequate levels of vitamin D during the neonatal period are described in Figure 1.

## DISCUSSION

The results showed inadequate maternal knowledge regarding the vitamin D metabolism and the risks and benefits of sun exposure in the neonatal period. The assessment of maternal knowledge is relevant as parents are the main people who are responsible for adequate habits of photoprotection, especially in early childhood, the most crucial moment for the interference of harmful effects of the sun on the development of the individual.^5^

A sample of 141 mothers was obtained. Most of them had had access to schooling, with 39.7% having had completed elementary school. When mothers were asked about exposing the baby to the sun during the first month of life, 93.6% responded that it was important, and 71.6% claimed they did it to improve their children’s vitamin D levels. Along the same lines, according to a study on mothers’ knowledge about sun exposure carried out in Sakarya, Turkey, the majority (87.5%) exposed their children despite being aware of the risks, the most frequent reason being the relationship between bone benefits and consequent vitamin D.^7^ So this habit is not exclusive to the region under study.

Assessing the forms of protection, 34.7% said they made use of some protection, the most mentioned means being sunscreen and clothing. Accordingly, a study carried out in the Netherlands also found a lack of parental knowledge regarding protection from sun exposure.^8^ Currently, in addition to the fact that there are no sunscreens recommended for newborns, the use of filters with high photoprotection values can interfere with the proper absorption of vitamin D — the initial reason for exposure — which demonstrates the amount of mistaken knowledge.^3^ Regarding time for sunbathing, 80.8% believe that the newborn can be placed in the sun for 10 to 30 minutes, and only two (1.4%) mothers denied the need for exposure, which they believed is correct. Thus, when comparing these results to the guidance given by the Brazilian Society of Pediatrics (SBP), which emphasizes that direct sun exposure in infants under six months should be avoided, it was observed that the knowledge demonstrated by the mothers goes against the adequate habits of photoprotection of the neonate.

Another important factor is lack of knowledge shown by mothers about the risks that early exposure to the sun can cause, since 76.6% denied that this habit in the neonatal period could cause any problems in the future, and among those who claimed it could cause some harm, only one mother mentioned skin problems. As mentioned before, the age at which direct exposure to sunlight begins is more important in determining skin cancer than the total lifetime exposure to sunlight.^4,5^ There is no safe level of exposure that can be recommended; therefore, it is necessary to consider that there must be a balance between sun exposure and the risks of skin cancer and other harmful effects that end up being recklessly ignored.^9^

When questioned, 81.5% of the mothers reported having heard about sun exposure improving vitamin D levels. However, when asked about the risks of hypovitaminosis D, 47.1% were unable to answer whether it could cause any health problem to the child, and among those that indicated possible risks, jaundice was the most cited. This shows a remarkable lack of knowledge of more than half of the mothers on the subject. Indeed, sun exposure favors an increase in vitamin D levels, and this in turn is of paramount importance in maintaining the homeostatic state of the body, but it is not the most suitable method for this purpose in the neonate period.^2^

Therefore, the erroneous belief that vitamin D is associated with a necessary sun exposure in the neonatal period becomes visible, confronting the current recommendations of the American Academy of Dermatology, which advises that vitamin D be obtained through diet and supplements alone, and emphasizes that sun exposure for this purpose is not recommended, especially in infants under six months, a population in which direct sun exposure should be strictly avoided.^4^ In contrast to this information, 68.1% of the mothers stated that isolated sun exposure is able to provide adequate levels of vitamin D for the newborn. To a certain point they are correct, but it would not be the healthiest way to obtain the vitamin in this period of life, and only 5% said that supplementation of vitamin D alone during the first month of life is necessary (Figure 1). Given the results obtained, it is possible to clearly observe the scarcity of knowledge by the mothers on the appropriate means of obtaining and supplementing vitamin D, as well as the relationship between sun exposure and its possible consequences during this period.

Another important point is the means of information through which these mothers have obtained the knowledge presented: 82.2% reported receiving medical advice to expose their baby to the sun during the first month of life, and 61% received this information with the purpose of improving the production of vitamin D. Such data reflect not only the unawareness of mothers on the subject, but also that of the medical population, bringing up a worrisome panorama. In the same perspective, a study carried out in Turkey showed that 45.7% of mothers also claimed to receive guidance and encouragement from health professionals with regard to sun exposure, demonstrating that this scenario is a reality not limited to the local environment, or even to a period or tradition.^7,10^

This study may have had some limitations regarding its population, with the possibility of reflecting regional knowledge, despite similar results having been obtained in international researches. It is worth noting that the population studied is predominantly coastal, which can affect habits. It would therefore be appropriate to carry out further studies in different regions in order to compare data. The study has a convenience sample and there is no sample size calculation. The questionnaire was developed by the authors with the purpose of answering the research question and it is not a validated tool.

In conclusion, our results indicate mothers’ inadequate knowledge regarding the acquisition of vitamin D and sun exposure in the neonatal period. The most alarming fact is lack of knowledge of the consequences of this inadequate early sun exposure, demonstrating an amount of inaccurate and erroneous information, mostly acquired through medical advice. This practice usually comes from local habits and customs perpetuated across generations. The need for further research into the subject is of paramount importance in order to take this information to all social spheres, reaching out to a larger number of families and professionals.
